# NFκB signaling in T cell memory

**DOI:** 10.3389/fimmu.2023.1129191

**Published:** 2023-02-24

**Authors:** Mark A. Daniels, Dezzarae Luera, Emma Teixeiro

**Affiliations:** ^1^ Department of Molecular Microbiology and Immunology, School of Medicine, University of Missouri, Columbia, MO, United States; ^2^ Roy Blunt NextGen Precision Health Building, School of Medicine, University of Missouri, Columbia, MO, United States; ^3^ Department of Surgery, School of Medicine, University of Missouri, Columbia, MO, United States

**Keywords:** NFκB signaling, T cell memory, protective immunity, immunological memory, environmental cues

## Abstract

Memory T cells play an essential role in protecting against infectious diseases and cancer and contribute to autoimmunity and transplant rejection. Understanding how they are generated and maintained in the context of infection or vaccination holds promise to improve current immune-based therapies. At the beginning of any immune response, naïve T cells are activated and differentiate into cells with effector function capabilities. In the context of infection, most of these cells die once the pathogenic antigen has been cleared. Only a few of them persist and differentiate into memory T cells. These memory T cells are essential to host immunity because they are long-lived and can perform effector functions immediately upon re-infection. How a cell becomes a memory T cell and continues being one for months and even years past the initial infection is still not fully understood. Recent reviews have thoroughly discussed the transcriptional, epigenomic, and metabolic mechanisms that govern T cell memory differentiation. Yet much less is known of how signaling pathways that are common circuitries of multiple environmental signals regulate T cell outcome and, precisely, T cell memory. The function of the NFκB signaling system is perhaps best understood in innate cells. Recent findings suggest that NFκB signaling plays an essential and unique role in generating and maintaining CD8 T cell memory. This review aims to summarize these findings and discuss the remaining questions in the field.

## 1 Introduction

The transcription factor Nuclear Factor kappa B (NFκB) regulates many aspects of the innate and adaptive immune system. Innate cells signal through NFκB to drive inflammation, while in lymphocytes, NFκB signaling supports their activation, differentiation, survival, and effector function. Perhaps due to NFκB’s varied and multiple effects, it has been challenging to dissect how and when NFκB signaling regulates each of these processes in T cells. Particularly in the context of the immune responses that accompany diseases such as autoimmunity, cancer, or infection. This review will focus on recent findings indicating that NFκB is critical for generating and maintaining T cell memory. The role of NFκB signaling in inflammation and lymphocyte activation has been recently reviewed in (1–3). While NFκB signals can regulate innate immune cells and immune responses and indirectly affect the differentiation of T cells to memory, here we will focus on the T cell-intrinsic mechanisms by which NFκB supports T cell memory and the environmental cues that drive them.

## 2 The NFκB signaling pathway

**Figure 1:**
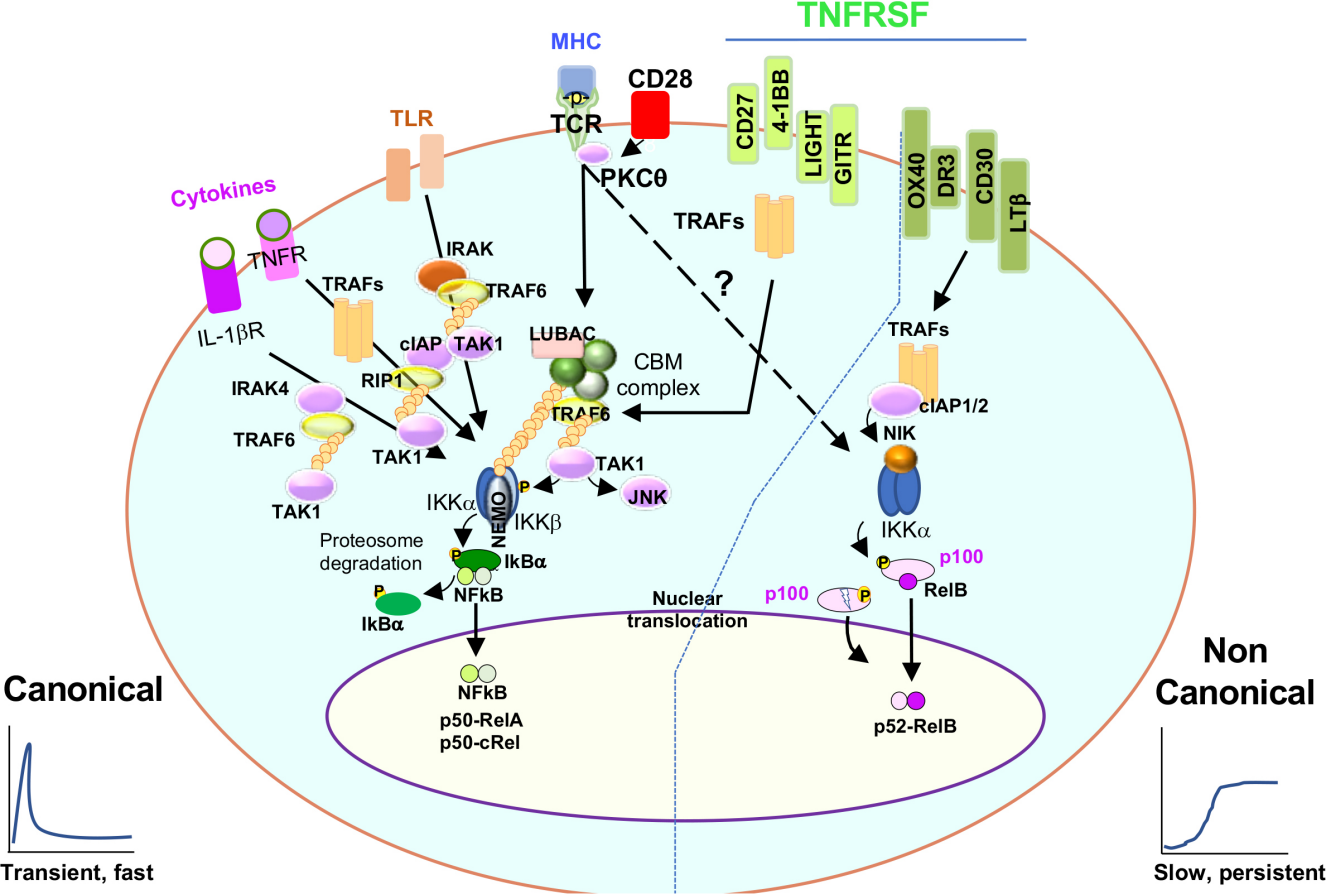
Canonical and non canonical NFκB signaling. The cartoon shows the multiple stimuli a T cell is exposed to and how they signal to NFκB. Discontinuous arrow indicates the TCR-dependent induction of non-canonical NFκB signaling. Refer to the text for more detail.

NFκB signaling is classically described as canonical and non-canonical. In T cells, recognition of antigen (Peptide-MHC) by the T cell receptor (TCR) and cluster of differentiation (CD) 28 (CD28) co-stimulation is required to activate the canonical NFκB signaling pathway. TCR engagement leads to the phosphorylation of the CD3 immunoreceptor tyrosine-based activation motifs (ITAMs) and recruitment of the zeta chain of T cell receptor associated kinase 70 (ZAP-70) to the plasma membrane, where it can, in turn, phosphorylate the adaptor molecules linker of activation for T cell (LAT) and SH2 domain-containing leukocyte phosphoprotein of 76kDa (SLP-76) enabling the assembly of the LAT/SLP-76 signalosome. Phosphorylated LAT binds to interleukin-2 inducible T-cell kinase (Itk) and phospholipase C γ1 (PLCγ1) and allows for Itk to phosphorylate and activate PLCγ1, which can then hydrolyze Phosphatidylinositol 4,5-bisphosphate (PIP2) in the membrane to generate two second messengers: inositol triphosphate (IP3), which binds to its Endoplasmic Reticulum receptor and triggers calcium signaling; and diacylglycerol (DAG) which is critical for the activation of protein kinase C θ (PKCθ) (3) (**Figure 1**).

PKCθ is a crucial enzyme for TCR-dependent canonical NFκB signaling. In resting conditions, PKCθ remains in the cytosol. However, upon TCR stimulation, SLP-76 phosphorylation allows for the recruitment of the guanine nucleotide exchange factor Vav, which helps to activate Rac GTPase and reorganize the cytoskeleton in a process that brings PKCθ from the cytosol to the membrane where, by the coordinated action of DAG, the GCK-like kinase (GLK) and 3-phosphoinositide-dependent protein kinase-1 **(**PDK-1), PKCθ is activated and connected to downstream signaling networks (CBM complex, explained below) (4–7). In the membrane, PKCθ is located in specific microclusters or lipid rafts where TCR and CD28 receptors are enriched. Of note, both antigenic and B7/CD28 signals are required for PKCθ activation, making PKCθ the first molecular hub where antigenic and costimulatory signals integrate to induce NFκB (8) (**Figure 1**).

The next link in the NFκB signaling cascade is CARD-containing MAGUK protein 1 (Carma1). Carma1 is a membrane scaffold protein recruited to lipid rafts after TCR stimulation. PKCθ, phosphorylates Carma1, which undergoes a conformational change and binds to another two molecules, B-cell lymphoma/leukemia 10 protein **(**Bcl10) and mucosa-associated lymphoid tissue lymphoma translocation protein 1 (Malt1), to form the CBM complex. The CBM complex acts next as a core where different adaptors, ubiquitin ligases, and enzymes ultimately activate the IκB kinase (IKK) complex (9, 10). In brief, activated Carma1 recruits the linear ubiquitin chain assembly complex (LUBAC) complex leading to the poly ubiquitination of Bcl10 and Malt1 *via* the action of the TNF receptor associated factor (TRAF) 6 ligase (TRAF6). This is followed by the activation of the Transforming growth factor-β (TGF-β)-activated kinase 1 (Tak1) and the recruitment of the IKK subunits (IKKα, IKKβ and IKKγ or NEMO). The proximity of Tak1 to the IKK subunits allows Tak1 to phosphorylate IKKβ. Phosphorylated IKKβ, phosphorylates the protein nuclear factor of kappa light polypeptide gene enhancer in B-cells inhibitor, alpha **(**IκBα), which is bound to the transcription factor NFκB in the cytosol. Finally, upon phosphorylation, IκBα is targeted for degradation in the proteasome, leaving NFκB free to translocate to the nuclei, where it regulates different aspects of gene expression[reviewed in (11) (discuss later) (**Figure 1**).

Non-canonical NFκB signaling is classically associated with tumor necrosis (TNF) receptor superfamily (TNFRSF) signaling. In resting conditions, NF-κB-inducing kinase (NIK) is continuously targeted for degradation upon the action of cellular inhibitor of apoptosis (cIAP) and TRAF2,3 ligase and adaptor activities. However, upon TNFRSF stimulation, cIAP mediates the K48 polyubiquitination and degradation of TRAF3. This allows for the accumulation of NIK and its activation, resulting in p100 processing, p52 production and binding to RelB (12) (**Figure 1**).

**Figure 2:**
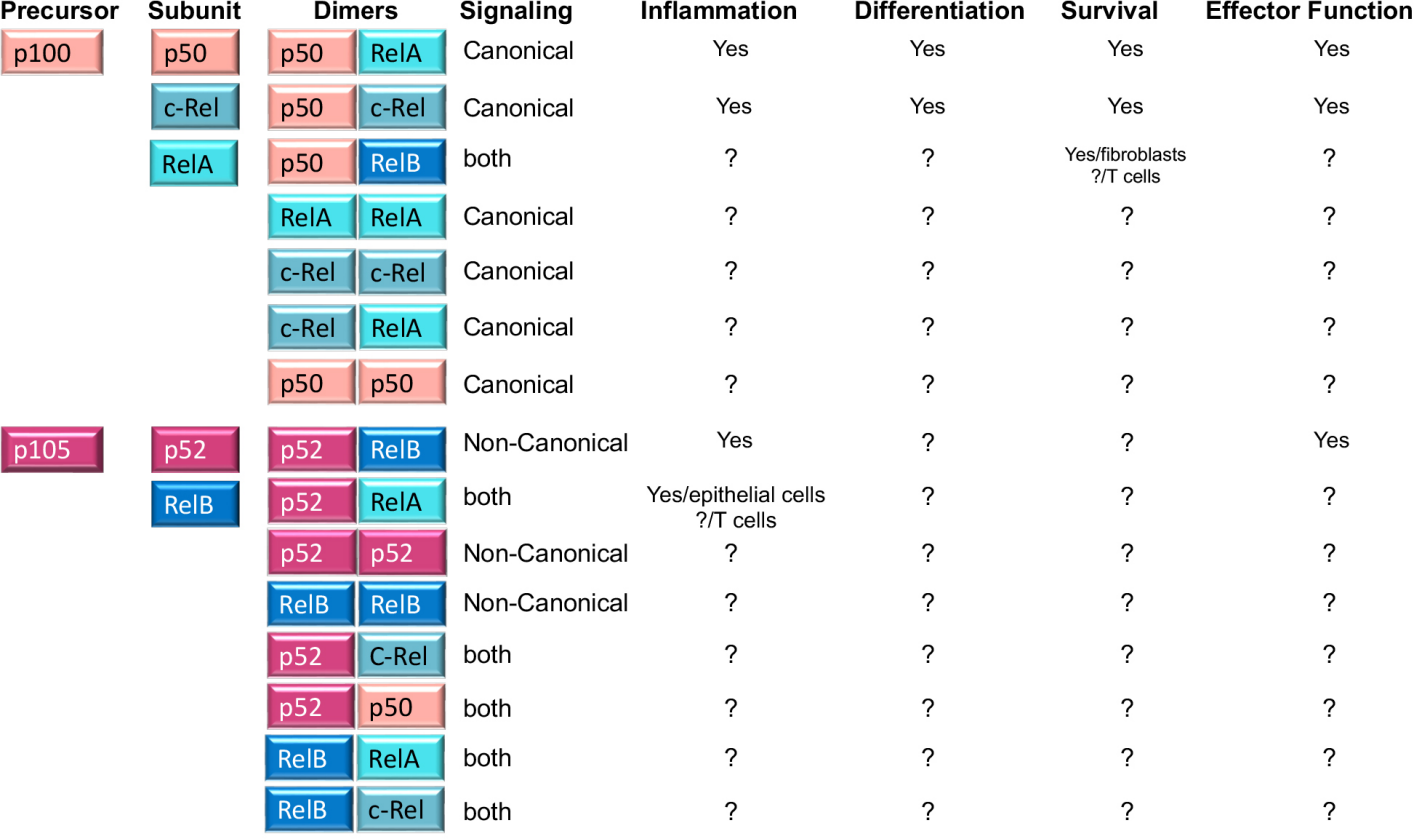
NFκB family members, homo and heterodimers and their roles in T cells. Second column shows the five NFκB subunits, p50, RelA, c-Rel, p52 and RelB that associate with each other to form fifteen potential dimers. Of these, three heterodimers (p50/RelA; p50/c-Rel and p52/RelB) are well known for their roles in T cell function and differentiation, while the role of the remaining five homodimers and seven heterodimers is unknown in T cells (indicated by? in the table). A role in other cells is indicated in the table. The table also shows whether any of the dimers is preferentially induced by canonical or non-canonical NFκB signaling (column 4) or whether it is the result of processing of precursor proteins p100 and p105 (column 1).

Importantly, NFκB comprises various homo and heterodimers whose composition differs between the canonical and non-canonical NFκB pathways. The canonical NFκB pathway involves p50/p65(RelA) and p50/c-Rel heterodimers, where p50(NFκB1) results from the constitutive degradation of p105 (13). In contrast to this, non-canonical NFκB signaling leads to the nuclear entry of the p52/RelB heterodimer, where p52 results from p100 processing. Another fundamental difference between canonical and non-canonical NFκB signaling is the composition of the IKK complex. While canonical signaling requires IKKβ, non-canonical NFκB signaling does not. Instead, the kinases NIK and IKKα are necessary to process p100 (the equivalent of IκBα) into p52 (12) (**Figure 2**).

It is widely accepted that antigen receptors do not trigger the non-canonical NFκB pathway (p100 processing and p52-RelB nuclear translocation). However, recent reports have challenged this view and shown that antigenic/TCR stimulation can also lead to non-canonical NFκB signaling (14–17). It is unclear whether the link between TCR and non-canonical NFκB signaling occurs directly or indirectly through TCR-dependent induced expression of TNFRSF members CD27, OX40, or 4-1BB (18). Contrasting this idea, Yu et al. showed that anti-CD3 stimulated T cells were able to induce non canonical NFκB members p100 and its mature form p52. Furthermore, they used NIK-deficient T cells and revealed that induction of p52 occurred much earlier than the time required for the induction of TNFR. These cells exhibited an activated phenotype that resembled effector and memory T cells (15). Non canonical NFκB signaling can also influence canonical NFκB signaling at least in two ways. On one hand by increasing the expression of RelA/RelA homodimers that are then activated by canonical signaling and can contribute to inflammation in the gut (19). On the other, by generating p52/RelA heterodimers that can reinforce RelA canonical NFκB activity (20). Both of these processes have been described in epithelial cells but a role in T cells remain yet to be explored.

Another fundamental difference between canonical and non-canonical NFκB signaling is the kinetics of their activation. While the canonical pathway leads to rapid but transient activation of NFκB (21–23), the activation of the non canonical pathway is slow and persistent (12). Finally, it is important to consider that crosstalk between both canonical and non-canonical pathways, not only in term of positive but also negative feedback loops,has been described (18) reviewed in (24, 25)), making it challenging to understand how this important signaling pathway regulates the many responses and differentiation processes of a T lymphocyte.

## 3 NFκB, T cell memory and T cell memory responses

**Figure 3:**
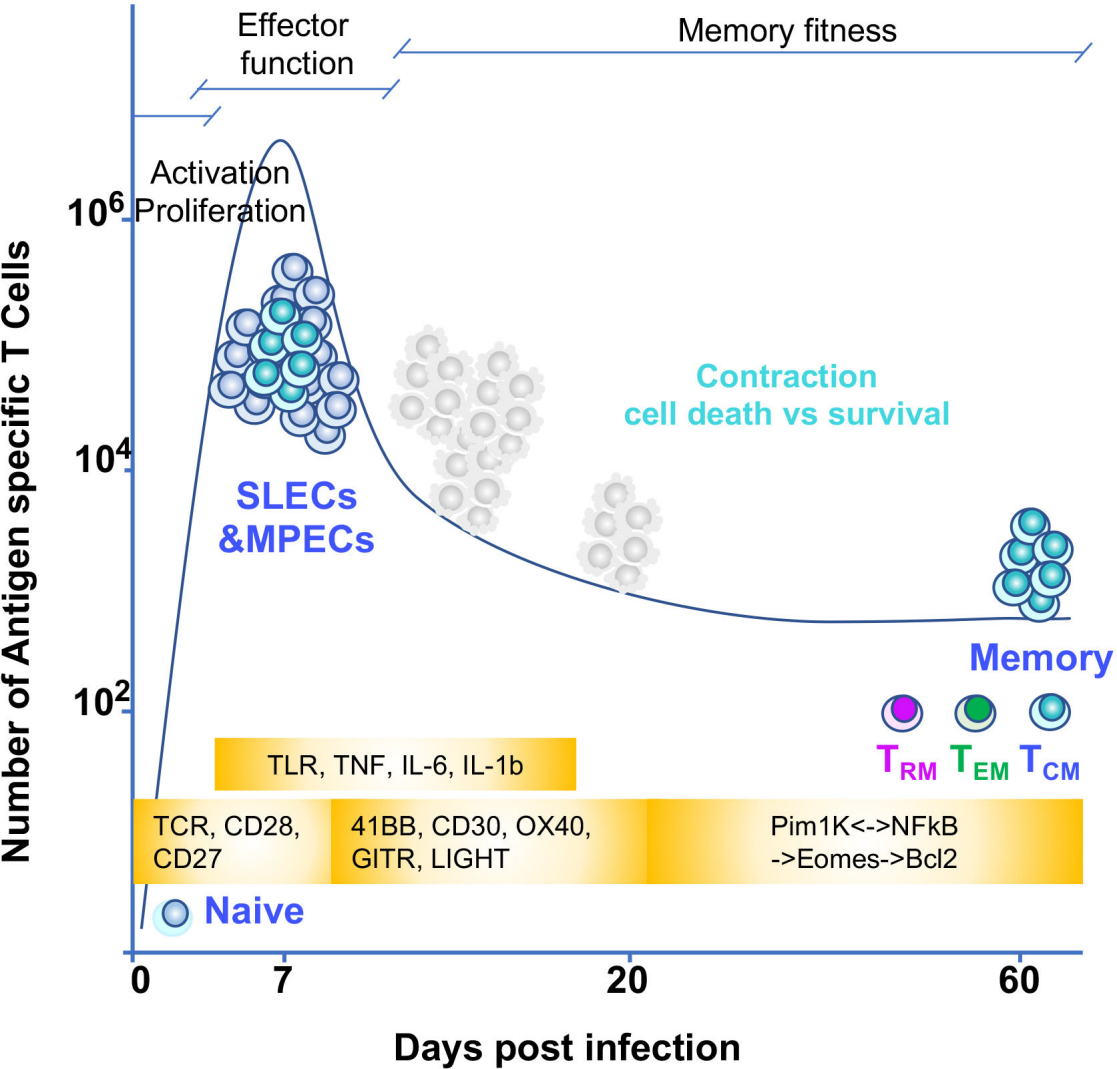
T cell memory differentiation and environmental triggers of NFκB signaling Naïve T cells recognize antigen through their TCR. TCR and CD28 signaling induce the NFκB pathway early in the response. This pathway is necessary for the cell to proliferate and acquire effector function. These signals together with other costimulatory signals from TNF receptors superfamily also signal to NFκB to sustain proliferation and effector function. In addition, inflammatory signals from cytokines and TLR could also contribute to shape the T cell intrinsic dynamics of NFκB signaling. Once effector T cells are generated they eliminate the cells source of antigen and inflammation and most of T cells (SLECs or short lived effectors) die by apoptosis. The MPECs or memory precursors continue their differentiation to memory. For this, an interplay between epigenetic and transcriptional changes allow for silencing of specific gene and poising others for quick effector gene reactivation. In addition, positive NFκB feedback loops allow for establishing memory fitness. The timing of these changes during T cell differentiation in relationship with a given T cell immune response are depicted in the figure through a graph of the kinetics of a T cell immune response to a hypothetical pathogen. Days of the immune response post infection are represented on the X axis. Y axis shows number of antigen specific T cells responding to the pathogen. Yellow blocks indicate the triggers of NFκB input over the course of the immune response.

During an immune response to pathogens, tumors, and self or transplanted organs, both naïve CD4 and CD8 T cells use their T cell receptors (TCRs) to recognize antigenic peptides associated with major histocompatibility complex (MHC) molecules (p-MHC) on the surface of dendritic cells (DCs) in secondary lymphoid organs such as lymph nodes or spleen. Antigen recognition, together with costimulatory and pro-inflammatory signals, provide the basic information that is required for a T cell to differentiate into effector and memory T cells. Effector and memory CD8 T cells are directly in charge of eliminating the cells that are the source of the antigen (i.e., infected or cancer cells) while CD4 effector and memory T cells indirectly contribute to the clearance of pathogens or tissues that are source of the antigen (via the activation or differentiation of other immune cells) (26, 27) (**Figure 3**). Both effector and memory CD4 and CD8 T cells do so through a series of changes in their epigenetic and transcriptional landscape that enable them to exert effector functions such as cytotoxicity or cytokine secretion. Yet a fundamental feature of memory T cells is their capacity to survive for long periods of time in the host and their ability to sustain their effector capabilities. The capacity to survive is a rare and unique characteristic in the responding T cell population since only 1-10% of the T cells recruited into the immune response can persist during the whole immune response and become memory (28) (**Figure 3**). Indeed, how memory T cells are generated and maintained it is still not fully understood. Importantly, memory T cells defend us against infection and cancer but also contribute to autoimmunity and transplant rejection. Therefore, thoroughly defining the mechanisms that regulate their generation and maintenance has important implications for immune therapies, including vaccines and immunotherapies targeting cancer, autoimmunity and transplantation.

The tremendous progress in the field in the last 20 years has given us a clearer idea of the transcriptional and epigenetic mechanisms that drive T cell memory. Since more is known about these processes in CD8 than CD4 T cells, from here on we will focus on CD8 T cells.

CD8 T cell memory is not monochromatic. Rather, CD8 memory is very diverse, comprising cells that stay in tissue (tissue-resident or T_RM_) and others that remain in the circulation, either in lymphoid organs (central memory or T_CM_) or in the vasculature as peripheral effector memory T cells (T_EM_) (29). This diversity promises to be even broader, with more specialized phenotypes being identified yearly (30, 31). Interestingly, each T cell memory subset’s survival and effector capabilities also appear to be distinct. For example, central memory T cells persist the longest and proliferate vigorously upon recall but are less efficient at effector responses upon recall than effector memory. By contrast, tissue-resident memory T cells in specific tissues, such as the lung, are short-lived but can quickly mount a vigorous response within the tissue (29).

In the search for biochemical mechanisms that explain how memory T cells arise, a part of the field has focused on how antigenic and inflammatory cues are transduced to signal the maturation toward memory. In this line, studies in the early 2000s determined mammalian target of rapamycin (mTOR) signaling was a major pathway used by inflammatory signals such as Interleukin-12 (IL-12) to limit the ability of effector T cells to progress to memory by increasing the ratio of transcription factors T-bet to Eomes (32). By contrast, Wnt/Phosphoinositide 3-kinase (PI3K) signaling turned out to be essential to favor the generation of memory T cells, in part by increasing the levels of tumor cell factor 1 (TCF-1) and Eomes and decreasing the T-bet/Eomes ratio (33–35). Another important finding was the realization that metabolic changes are also necessary for T cells to become memory and that these changes were modulated by the intensity of antigenic and inflammatory signals (36–39).

NFκB is a pleiotropic signaling pathway with important roles in controlling inflammation, cell activation, and cell survival. Studies by Schmidt-Supprian, Rajewsky and Pasparakis determined the crucial relevance of NFκB signaling to maintain the survival of naïve T cell (40). Multiple studies also established that NFκB signaling is required for early T cell activation, cell division, cytokine production, and cytotoxicity (2). Using animal models whose T cells were devoid of several signaling intermediates in the NFκB signaling cascade, studies found that this pathway is especially relevant for the differentiation and/or effector function of CD4 T helper (Th) Th1, Th2, Th17, Tfh and regulatory T (Treg) cell subsets (1, 41–43). The role of NFκB in T cell memory has been less explored, perhaps because NFκB is critical for most of the early aspects of T cell activation, proliferation, and effector differentiation, making it challenging to dissect its unique roles in T cell memory. One study, however, reported that CD8 T cells uniquely impaired in triggering TCR-dependent NFκB signals failed to differentiate into memory T cells in response to infection. The memory defect correlated with impaired RelA and c-Rel transcriptional activity. Yet, these NFκB-impaired CD8 T cells did not exhibit any defects in proliferation or effector function.This work indicated that the antigenic signals could control CD8 T cell memory through NFκB (44). Follow-up studies determined that inhibiting T cell-intrinsic NFκB signaling exclusively after the peak of the immune response to *Listeria monocytogenes* infection (once T cells have acquired effector function) dramatically hindered the ability of T cells to express the memory-associated transcription factor Eomes (45, 46). As a consequence, CD8 T cells failed to transition to memory. This was true for memory T cells in circulation, with inhibition of NFκB affecting mainly memory T cells of a central memory phenotype. Interestingly, the study also suggested that once memory T cells are formed, they also depend on NFκB signaling for their maintenance, as pharmacological inhibition of NFκB decreased B-cell lymphoma 2 (Bcl2) expression and led to a loss in memory fitness (46).

It is still poorly understood whether NFκB regulates CD4 T cell memory in the same manner or if its requirement will differ depending on the specific T-helper subset. Likewise, the potential role of NFκB in the differentiation of resident memory T cells has remained elusive. Using inducible NFκB models, we have recently described that lung tissue-resident memory T cells need a continuous input of NFκB signals for their maintenance. Inducible activation of NFκB signals in influenza-specific lung CD8 T_RM_ cells boosted their numbers, presumably by increasing CD122 or IL15Receptor B chain and Bcl2 levels. This also appears to be true for circulatory T_CM_ and T_EM_ cells. Strikingly, we found that the role of the NFκB signal in generating lung CD8 T_RM_ (before the T_RM_ pool has been established) is different and unique. Inducible activation of NFκB signals in CD8 T cells transitioning to T_RM_ led them to succumb. In contrast to this, T cell-intrinsic inhibition of NFκB signaling resulted in a considerable increase in the generation of influenza-specific CD8 T_RM_ in the lung upon influenza infection. Remarkably, the same manipulation of NFκB signaling had the opposite effect on the generation of T_CM_ and no effect on T_EM_ (47). These results are surprising and show that NFκB signaling is a regulator of T cell memory subset diversity. Most importantly, these studies offer new avenues to boost T_RM_ in the lung. This is especially relevant in the case of respiratory infections, and its vaccines as CD8 T_RM_ are known to be short-lived and are indispensable for heterosubtypic protection (48).

While it may take days for naïve T cells to generate an immune response, memory T cells can start secreting cytokines and eliciting their effector function in a matter of hours. Studies by the Farber’s group have shown that inhibition of NFκB activity prevented early memory T cell signaling and TCR-mediated effector function, suggesting that NFκB is required for early recall responses (49). Speed is not the only component of a healthy T cell memory response; the location of these cells also plays a role in the protection they provide. Another study showed that mice receiving lung memory T cells exhibited a rapid and enhanced viral clearance compared to those receiving spleen memory cells or naïve mice (50). Thus, maintaining T_RM_ in tissue and their functions is crucial to provide long-term immunity, although whether the latter depends on NFκB signals remains to be determined. Next, we will discuss how different environmental cues required for the generation of T cell memory may modulate the levels of T cell-intrinsic NFκB signaling and their fate.

## 4 Extracellular signals that trigger NFκB signaling and can shape T cell fate 

Naïve T cells circulate between the lymph and blood, passing by secondary lymphoid organs (lymph nodes and spleen). They use their TCR to scan other cells’ peptide-MHC molecules and detect the presence of foreign antigens they have not been tolerized against. This detection is based on interactions of TCR with peptide-MHC and triggers different signaling pathways (including NFκB) that are critical to ‘prime a T cell’ for activation, proliferation, and secretion of IL-2. Once a T cell is activated, it leaves the secondary lymphoid organ where it first encountered antigen and travels through the circulatory system to tissues where it can finally exert its true effector functions (**Figure 3**). Already at priming and later, when in tissue, a T cell can continue receiving antigenic/TCR signals together with other signals, such as costimulation *via* CD28 or TNFR superfamily members (such as CD40/CD40L, OX40, 4-1BB, Herpes Virus Entry Mediator (HVEM), or CD27). In addition, some inflammatory cytokines secreted by other cells in tissue can bind to receptors on T cells and signal to NFκB. Finally, T cells can also express and signal through TLRs (51). Some of these molecules are known for activating the canonical NFκB signaling pathway. However, others use the non-canonical NFκB signaling pathway (51, 52) (**Figure 1**). Yet how a T cell integrates all these NFκB signal inputs across time or depending on the T cell differentiation status is unclear. Likewise, how a T cell interprets differences in dose, duration, and amplification of these NFκB inputs is a complex issue that remains largely unknown. It is also unclear whether previous stimulatory events are preserved in a T cell’s molecular memory and pre-conditioning its subsequent response to the same stimuli as it has been recently reported for fibroblasts (53). Next, we will discuss in more detail the main environmental signals that can support T cell-intrinsic NFκB signaling as a T cell matures to memory.

### 4.1 Tumor necrosis factor receptor superfamily

Members of the TNFR superfamily signal through NFκB mainly through the canonical pathway (54). CD30, Lymphotoxin αβ (LTαβ), B- and T-lymphocyte attenuator (BTLA), CD160, OX40, 4-1BB, CD27, Glucocorticoid-induced TNFR-related protein (GITR), death receptor 3 (DR3), and HVEM expression are induced on the surface of T cells once they have become activated (54, 55) (56), and their expression can be maintained for hours or even days[review in (54)]. When they interact with their ligands (which are expressed on DCs, B cells, or macrophages (57–59) or non-immune cells in response to inflammation), these receptors can positively regulate CD4 and CD8 T cell responses and affect the outcome of disease (60). The consequences of TNFR-ligand interaction are bidirectional, as cells expressing the ligand also become activated. Given that T cells can express both receptors and ligands, this suggests that T cells within a population can potentially influence each other’s responses (61).

In general, TNFRSF members regulate T cell responses in two ways. On one side, they support the production of cytokines directly on the T cell or indirectly by inducing the secretion of pro-inflammatory cytokines by antigen presenting cells (APCs). On the other side and most relevant to this review, TNFRSF members regulate the frequency of effector and/or memory T cells generated after antigen priming both in primary and recall immune responses. They do so *via* NFκB by regulating both the proliferative and survival capacities of the T cell [review in (54)]. This has been described for 41BB, CD27, TNFSF14 or LIGHT and GITR (62–67), which support the expression of anti-apoptotic factors B-cell lymphoma-extra large (Bcl-xL), Bcl2 and BFL-1 (68). Most recently, 4-1BB has also been shown to modulate T cell mitochondrial metabolism to promote survival and persistence of memory T cells (69, 70).

### 4.2 Cytokines that signal through NFκB and their effects on T cell memory

Inflammatory cytokines, TNF, interleukin 6 and 1 β (IL-6, and IL-1β) levels increase over the course of infection in specific tissues where pathogen-specific T cells are present. T cells express receptors that can bind to these cytokines and, in this manner, increase the input of signals to NFκB. As a pro-inflammatory cytokine, TNF is known for inducing apoptosis in highly activated effector T cells. T cells, themselves, can express membrane-bound TNF, although this form of TNF needs to be cleaved to be most effective (71). T cells also express TNF receptor 2 (TNFR2) and can interact with TNF early upon antigen recognition. In this way, TNF acts as a costimulatory signal and can lower the threshold of TCR signaling to support proliferation, survival, and cytokine production *via* NFκB (72, 73). TNF can also induce apoptosis on highly activated T cells, such as effectors, during and after the peak of an immune response (the contraction phase- **Figure 2**). For this, TNF needs to interact with TNF receptor 1 (TNFR1) on the surface of T cells. Curiously, apoptosis of T cells during the contraction phase of the response to Lymphocytic choriomeningitis virus (LCMV) is impaired when T cells lacked both TNF receptors, TNFR1 and TNFR2 (74). By contrast, selective deletion of TNFR2 appears to enhance the persistence of CD8 T cells maturing to memory (75). These findings suggest a delicate balance between TNFR1 and TNFR2 signals regulates T cell memory.

IL-1β triggers the canonical NFκB pathway and can also modulate T cell responses. T cells can indeed express IL-1 receptor (IL-1R), and when they bind IL-1, this interaction can enhance T cell proliferation and effector responses of both naïve and memory T cells (76, 77) (**Figure 1**).

IL-6 has also been shown to induce NFκB signaling in intestinal epithelia (78), although whether IL-6 signaling in lymphocytes also induces NFκB remains to be defined. Most recently, the Medhitovz group has shown that IL-6 and IL-1 cooperate to control the inhibitory effects of regulatory T cells on CD4 T cells and that IL-6 is critical for the generation of memory CD4 T cells. Yet whether this is dependent on NFκB signaling, relates to T cell survival, or has a similar effect on memory CD8 T cells is still unclear (79).

In summary, cytokines are often considered major Janus kinase (JAK)-signal transducer and activator of transcription (STAT) pathway (JAK/STAT) signaling users to shape CD4 T helper differentiation. However, a few of the inflammatory cytokines (IL-6, IL-1, and TNF) present in abundance in disease scenarios such as infection, chronic inflammation or therapies such as chimeric antigen receptor (CAR) immunotherapies (80) can also limit the generation of long-term protective T cell memory and may do so through NFκB signaling. The ability to trigger NFκB signals makes them unique as they could contribute to shaping the overall NFκB system of the cell and alter its ultimate fate.

### 4.3 Toll like receptors

TLRs are pattern recognition receptors that recognize microbial molecules characteristic of specific pathogens. They are expressed in human and mouse sentinel cells such as DCs and macrophages. TLRs are comprised of 13 different receptors (TLR1-13), some of which are not shared by humans and mice, and some are expressed upon TCR stimulation (51). TLRs classically trigger canonical NFκB signaling *via* Tak1 (52, 81). Upon ligand binding, TLRs dimerize, and their TIR domains are recognized by receptor-proximal membrane molecules The Toll-interleukin-1 Receptor (TIR) domain-containing adaptor protein (TIRAP) or TRIF-related adaptor molecule (TRAM), which enable the assembly of two different scaffold supramolecular organizing centers (SMOCs). TIRAP seeds the Myddsome while TRAM seeds the Triffosome. The Myddsome includes Myeloid differentiation primary response 88 (MyD88) and Interleukin-1 receptor-associated kinase (IRAK) kinases, which, once activated, recruit the ubiquitin ligase TRAF6. TRAF6 mediates the activation of Tak1, thereby inducing NFκB signaling. TRAF6 also mediates the activation of TANK-binding kinase 1 (TBK1), which can also feedback on NFκB signaling and activate glycolysis *via* protein kinase B or Akt. (reviewed in (82) (**Figure 1**).

TLR2, TLR5, and TLR9 work as costimulatory signals on effector T cells, but only when antigen recognition is in place. As co-stimulatory molecules, they support proliferation and effector function (51). Instead, T cell-intrinsic TLR2 signaling has recently been shown to regulate CD8 T cell survival and CD8 T cell memory. In the context of viral infections, TLR2-Myd88 deficient CD8 T cells poorly differentiate into memory T cells due to an inability to survive during the contraction phase of a vaccinia virus infection (83). The authors suggested that this depended on PI3K activity although a contribution of NFκB signaling still remains to be explored (83). Another study showed that CD8 T cells stimulated with low-affinity antigen and TLR2 ligands more efficiently generated memory phenotype CD8 T cells than without TLR costimulation. These memory-type CD8 T cells could respond and survive *in vivo (* 84). While less is known for other TLRs, it is tempting to hypothesize that TLR can have an important role in modulating T cell memory either directly depending on the signal input the T cell receives or indirectly *via* the regulation of the function of other immune cells.

## 5 Intracellular signals that feed into NFκB signaling and can shape T cell fate

NFκB signaling can also be induced in a T cell without involving extracellular receptors. An obvious example is intracellular TLR3,7, and 9, which are expressed on endosomal membranes and signal to NFκB (52). Yet aside from TLR, other innate sensors able to signal to NFκB have been recently shown to be expressed inside T cells. The one that has drawn most attention recently is Stimulator of interferon genes(STING). STING is an adaptor molecule that binds to cyclic di-nucleotides generated by intracellular pathogens or cyclic guanosine monophosphate (cGMP) generated as a consequence of the activation of the Cyclic GMP–AMP synthase (cGAS) once it has recognized cytosolic DNA. STING, together with TLRs, supports the activation of NFκB and interferon β (IFNβ). For STING signaling, the recruitment of TBK1 to STING allows both the activation of the IKK complex and induction of NFκB and the activation of interferon regulatory factor 3 (IRF-3) (85). We have recently described that T cell-intrinsic STING signaling can tightly regulate CD8 T cell memory (86). During the course of a T cell immune response, we found that excessive STING signaling hindered the generation of CD8 T cell memory but this was reverted when T cells lacked STING. Our studies also found that TCR signal strength regulated the level of induction of STING signaling within effector CD8 T cells when they were exposed to STING agonists. Strong antigenic signals supported the maximum level of STING signaling when T cells were exposed to STING agonistic stimulation. In turn, high levels of STING signaling upregulated the expression of the integrated stress response (ISR) molecule C/EBP homologous protein (CHOP), which caused Bcl-2 Interacting Mediator of cell death (Bim)-mediated apoptosis in T cells transitioning to memory (86). We believe these findings are especially important in the context of current STING agonistic therapies developed for cancer treatment. They suggest tumor-specific T cells may be lost if STING agonist levels are not properly dosed. The data also has important implications for the treatment of infections that cause persistent activation of STING or inflammatory diseases where STING signaling is aberrantly overactivated (87–90). In these cases, treatments that attenuate STING signaling might be especially critical to regulate the deleterious effects of inflammation and to preserve the generation of T cell protective immunity (91).

It is known that NFκB signaling can be subjected to negative feedback loops *via* the regulated expression of inhibitors IκBα, IκBδand IκBε (92, 93). These negative feedback loops establish a different signaling modality from the typical transient/strong or delayed/sustained NFκB activation. They introduce the idea of oscillation which can be interpreted by the cell as a completely different input using the same signaling pathway (94). Little is known, however, of positive feedback loops that can perpetuate signaling without external input. It has been proposed that positive feedback loops in a signaling network (or bi-stability) can create a memory of a transient differentiating stimulus long after the stimulus is removed by establishing self-sustaining patterns of gene or protein expression (95), thereby providing a framework of how cell differentiation can be achieved and lineage commitment can be maintained (96). Most recently, such a positive feedback loop has been shown for NFκB signaling in the context of T cell memory (46). One study described a novel mechanism of how commitment to CD8 T cell memory could be achieved by perpetuating NFκB signals *via* a feedback loop long after the original TCR-dependent NFκB signaling had been removed. In their study, the authors reported how the TCR-dependent NFκB signal delivered early in the immune response led to the expression of a kinase, Pim-1 proto-oncogene, serine/threonine kinase (Pim1K), that was essential to maintain the induction of NFκB signals late in the response while T cells were differentiating to memory. Impairing the activity of any of the members of the feedback loop resulted in a loss of the memory T cells generated upon infection, indicating that this NFκB positive feedback loop is critical to T cell memory fitness (46).

Taken altogether, it is becoming clear that during the course of an immune response, T cells are subjected to a myriad of extracellular and intracellular inputs that feed into the NFκB signaling system. These inputs might be wired in different ways to generate distinct responses and a memory of the original stimuli that is perpetuated to enable T cell differentiation and final fate commitment (53).

## 6 NFκB signaling and T cell memory transcriptional and epigenetic programming

The NFκB signaling pathway leads to the induction of the transcription factor NFκB. As mentioned before, NFκB is a dimer that can be composed of the combination of two of five different subunits, p65(RelA), p50 (NFκB1), c-Rel, p52 (NFκB2), and RelB (**Figure 2**). All subunits share a rel homology binding domain (RHD) necessary for DNA binding and binding to other subunits. In addition, each subunit contains transactivation domains responsible for transcriptional activity[review in (42)]. Much is known about the signaling intermediates that lead to NFκB nuclear translocation and the external stimuli that support this process, but how NFκB regulates endogenous target genes has remained elusive. Similarly, why and how NFκB leads to different gene expression patterns depending on the cell type has yet to be elucidated.

Two main features (or outcomes here) define a memory T cell. On one side, the ability to quickly re-activate effector functions. On the other side, the unique ability to have a long life. Resolving how T cell memory is regulated involves understanding whether the same mechanisms control these two features or not. In the case of T cell memory, it is important to consider that the genetic program a memory T cell inherits comes from previously activated effector T cells. Add to this the idea that T cell memory differentiation continues long after antigen is cleared, and the possibility to integrate environmental cues from tissue, and it is easy to perceive the level of complexity in memory programming.

A way that may help to tease this apart is to consider that the capacity to be long-lived and the capacity for quick induction of effector function can be differentially regulated by NFκB. If we consider the transcription factors that are now well known for their ability to regulate T cell memory differentiation[recently reviewed in (97)], one can observe that some have a more significant role in memory survival than in effector function differentiation. For example, in the T-bet/Eomes tandem, increasing Eomes levels over T-bet is necessary to support Bcl-2-dependent survival (98). In the B lymphocyte-induced maturation protein-1(Blimp-1)/(B-cell lymphoma 6)Bcl-6 tandem, however, Blimp-1 is more involved in effector function and Bcl-6 in memory fitness. NFκB regulates both Eomes and Blimp-1. Yet, TCR-dependent NFκB signaling deficient CD8 T cells, are deficient in Eomes but not in T-bet or Blimp1 expression. They can differentiate into effectors but fail to progress to memory (46), indicating that TCR-dependent NFκB signals uniquely utilize NFκB signaling to program the longevity of memory T cells. Interestingly, NFκB directly regulates the expression of Eomes (99) but the NFκB -dependent regulation of Blimp-1 may follow a different mechanism. Thus, establishing a program of memory longevity might involve the transcriptional ability of p65 NFκB alone or with specific players such as Notch to regulate Eomes expression (99, 100) but the same mechanisms may not control Blimp-1.

When considering the different T cell memory subsets, one can only wonder whether the same NFκB mechanisms apply to all. Eomes and TCF-1 are master regulators of central memory T cells in the circulation. However, too much Eomes expression and too little T-bet expression prevents T cells from becoming tissue-resident memory (97). Instead, the induction of the transcription factor Runx3 appears more crucial for T_RM_ homeostasis (101). In this case, Eomes may be dispensable for T_RM_ survival although NFκB signaling may still have a role in CD8 T_RM_ by regulating in a different manner Runx3 expression. One possibility is *via* the Pim1K axis as Pim1K is required for Runx3 nuclear translocation (46, 102).

**Figure 4:**
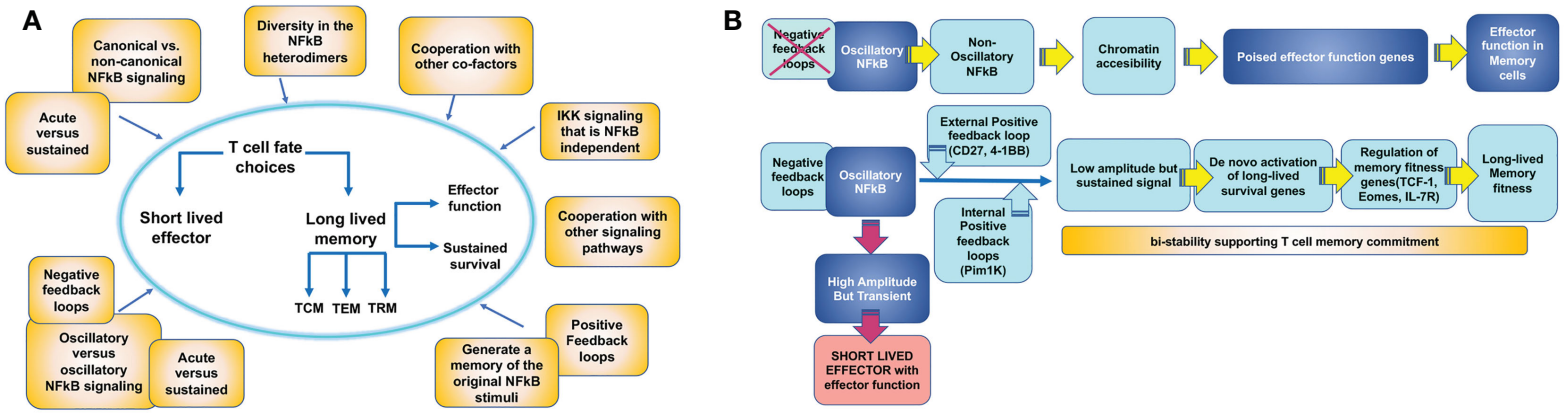
Mechanisms by which T cell intrinsic NFκB signaling could contribute to establish T cell memory outcome. **(A)** An activated antigen specific T cell acquires effector function and can either choose the fate of a long-lived memory T cell or die as a short lived effector T cell (T cell fate choice). Becoming a memory T cell involves the ability of rapid re-activation of effector functions and sustained survival. These two features may be regulated independently by NFκB signaling. Meanwhile, memory T cell precursors also differentiate into different T cell memory subsets: circulating central memory (T_CM_) and effector memory (T_EM_) cells or tissue resident memory (T_RM_) cells. The three subsets may be regulated independently by NFκB signaling. Processes that can contribute to determine these T cell memory choices are illustrated in yellow boxes. **(B)** Model for how NFκB signaling could regulate memory effector function and memory survival through different mechanisms.

Other mechanisms may be also in place to regulate other molecular players of T cell memory and explain how NFκB signaling leads to different T cell fates (**Figure 4**). Some T cell outcomes may be determined using the canonical versus non-canonical pathway and the induction of specific NFκB heterodimers (**Figure 2**). Additionally, the accessibility of NFκB to specific promoters depends on the cell’s open vs. closed chromatin status (103, 104). The ability of NFκB to synchronize with other co-factors may also contribute to specific gene expression in T cells differentiating to memory (105). Finally, the dynamics of the NFκB response can vary depending on the frequency and type of extracellular signal input, which might be translated by the cell into different transcriptional responses or no response at all. For example, NFκB signals can be oscillatory as long as a stimulus can produce waves of NFκB nuclear entry under the NFκB-dependent expression of the IκBα feedback loop. Alternatively, NFκB oscillations may be dampened by the expression of alternative IκB inhibitors that are not susceptible to NFκB control. Oscillatory and dampened NFκB signal modes have been shown to regulate different genes depending on mRNA half-life and chromatin-regulated mechanisms (104, 106, 107). Although this has not been explored, it is possible that some of the extracellular cues a T cell samples in circulation or tissue can deliver distinct NFκB nuclear dynamics, thereby explaining why only certain NFκB -dependent genes will be expressed. Another possibility that could explain NFκB -dependent T cell outcome diversity is the unique contribution of the two transactivation domains of the p65 NFκB subunit depending on posttranslational modifications as has been recently described for primary fibroblasts (108).

In summary, NFκB is a mediator of a complex signaling circuit able to integrate multiple environmental cues over time and interpret them into specific T cell outcomes, including T cell memory (**Figure 4**). However, a thorough understanding of how these mechanisms operate in T cells is still missing. Pioneering work using mathematical models and system approach strategies is revealing some of these mechanisms for innate cells (53, 109–111). These exciting studies may have drawn out the map to solve the unresolved questions of how NFκB regulates T cell outcome.

## 7 Alternative IKK/NFκB signaling triggered by environmental cues

While it is often assumed that the NFκB signaling cascade exclusively induces the transcriptional activation of NFκB, increasing evidence demonstrates that intermediates in this pathway can alternatively lead to the activation of other signaling pathways and the induction of transcription factors in an NFκB-independent manner. Already when PKCθ function was described, it was evident that it could regulate both NFκB and MAPK activities. More recently, other signaling intermediates of the NFκB pathway, such as Carma1, have been shown to activate mTOR upon TCR stimulation (112). Malt1 and IKKs can activate MAPKs independent of NFκB induction (11, 113). In addition, environmental cues, such as cytokines and TLRs also use the NFκB machinery to trigger activation of other specific Mitogen-activated protein kinases **(**MAPK) signaling pathways (114–116).

There is little understanding of how alternative NFκB signaling modes regulate T cell responses and memory. Even less is known of whether the IKK/NFκB signaling pathway can be selectively directed to only support these alternative pathways and not NFκB transcriptional activation or vice versa, depending on the signal input from cytokines, antigens, or TLR ligands.

We propose a comprehensive integration of all of the ‘flanks’ of the NFκB signaling pathway is necessary to fully understand how this essential signaling pathway regulates T cell-mediated immunity and to include this knowledge into therapies that lead to better vaccines and T cell-based immunotherapies.

## 8 Conclusion

NFκB signaling is often associated with inflammation. Naïve T cells depend on the NFκB signal for their survival, while recently activated T cells proliferate and secrete IL-2 and interferon γ (IFNγ) in an NFκB -dependent manner. Most recently, it has been shown that NFκB signaling is a crucial signaling pathway for generating and maintaining CD8 memory T cells. NFκB signaling appears especially critical for antigenic signals to program memory fitness and maintenance even after infection has resolved. Yet there are still important gaps that need solving. Besides antigen, other inflammatory signals present during an immune response can signal to NFκB within a T cell. It will be important to define how and which aid in generating and maintaining T cell memory. We also do not clearly understand whether the level of NFκB signal a T cell could experience in the context of disease shapes T cell memory in different ways. Understanding this can be critical for the treatment and/or vaccination of patients either suffering from chronic inflammation or being subjected to anti-NFκB therapies t. Lastly, the field is still in its infancy in understanding how NFκB signaling can specifically regulate T cell outcomes. It is becoming increasingly clear that a more systemic approach based on mathematical models and foundational work in innate cells needs to be applied to T cells to better define how this signaling pathway can be exploited therapeutically to shape T cell-based protective immunity.

## Author contributions

ET wrote and edited the manuscript as well as organized the review. MD edited and contributed to the discussion of the manuscript. DL contributed to the organization and discussion of the manuscript. All authors contributed to the article and approved the submitted version.
